# The relationship of ALPK1, hyaluronic acid and M1 macrophage polarization in the temporomandibular joint synovitis

**DOI:** 10.1111/jcmm.18172

**Published:** 2024-03-17

**Authors:** Jie Zhao, Yaping Feng, Xin Liu, Huimin Li, Huilin Guo, Jin Ke, Xing Long

**Affiliations:** ^1^ State Key Laboratory of Oral and Maxillofacial Reconstruction and Regeneration, Key Laboratory of Oral Biomedicine Ministry of Education, Hubei Key Laboratory of Stomatology, School and Hospital of Stomatology Wuhan University Wuhan China; ^2^ Department of Oral and Maxillofacial Surgery, School and Hospital of Stomatology Wuhan University Wuhan China

**Keywords:** ALPK1, hyaluronic acid, macrophage polarization, PKM2, temporomandibular joint synovitis

## Abstract

M1 macrophage polarization and synovitis play an important role in the pathogenesis of temporomandibular joint osteoarthritis (TMJOA). Reduced molecular weight of hyaluronic acid (HA) in synovial fluid of patients with TMJOA. In addition, high molecular weight hyaluronic acid (HMW‐HA) is often used clinically to treat TMJ inflammation. As a pattern recognition receptor of the cytoplasm, ALPK1 was found to be pro‐inflammatory in a variety of diseases. However, the relationship of ALPK1, HA and M1 macrophage polarization in TMJ synovitis remains unclear. We aimed to investigate the role of ALPK1 and HA in macrophage polarization and TMJ synovitis and the underlying mechanisms. The results demonstrated that ALPK1 was highly upregulated in the synovial macrophages in the inflamed TMJ synovium of patients. Low molecular weight hyaluronic acid (LMW‐HA) promoted the expression of ALPK1 and M1 macrophage‐associated genes. Besides, rhALPK1 promoted the expression of M1 macrophage‐associated factors and the nuclear translocation of PKM2. Furthermore, ALPK1 knockout mice exhibited limited infiltration of macrophages and decreased expression levels of M1 macrophage‐associated genes in CFA‐induced TMJ synovitis. While HMW‐HA inhibited the expression of ALPK1 and M1 macrophage polarization. Our results elucidated that ALPK1 promoted TMJ synovitis by promoting nuclear PKM2‐mediated M1 macrophage polarization, whereas HMW‐HA inhibited the expression of ALPK1 as well as M1 macrophage polarization.

## 1 INTRODUCTION

Increasing evidence indicates that synovitis plays an important role in the development of temporomandibular joint osteoarthritis (TMJOA) and may be a precursor to TMJOA.^1^, ^2^ It has been confirmed that TMJ synovium was mainly composed of synovial macrophages and synovial fibroblasts.^3^ A significant increase of macrophages was discovered in the inflamed synovium.^4^, ^5^ Macrophages can be divided into classically activated M1 macrophages and alternatively activated M2 macrophages in response to the immune microenvironment.^6^ There is a dramatic increase in the number of M1‐type macrophages in the inflamed synovium.^7^ Studies have shown that M1 polarization of synovial macrophages aggravates CFA‐induced experimental TMJ inflammation by producing a large number of pro‐inflammatory cytokines including IL‐1β, IL‐6 and TNF‐α.^8^, ^9^

ALPK1 is a pattern recognition receptor of cytoplasm for ADP‐heptose.^10^ A series of studies have demonstrated that ALPK1 plays an important role in the occurrence and development of various inflammatory diseases.^11^, ^12^ ALPK1 could promote the expression of TNF‐α and IL‐8, and inhibiting ALPK1 could effectively inhibit inflammation.^13^, ^14^ A study found that the expression of ALPK1 was positively correlated with macrophage infiltration in renal tissue.^15^ Faass, L. et al. found that pure ADP‐heptose could promote the activation of THP‐1(a human acute monocytic leukaemia cell line) cells and human primary macrophages, while ADP‐heptose and bacterial enzyme‐treated lysates could promote the differentiation of monocytes into M1 macrophages.^16^ Concerning that ADP‐heptose functions following the combination of ALPK1,^17^ it is reasonable to hypothesize that ALPK1 may regulate M1 macrophage polarization to participate in synovial inflammation of TMJ.

PKM2 is a key rate‐limiting enzyme for glycolysis. Recent studies have shown that PKM2 plays an important role in the M1 macrophage polarization.^18^ The monomers and dimers of PKM2 can enter the nucleus to promote glycolysis and inflammatory responses, and the tetramer of PKM2 in the cytoplasm can mediate the tricarboxylic acid cycle, oxidative phosphorylation and anti‐inflammatory responses.^19^

HA is a normal component of TMJ synovial fluid, which has important effects such as lubrication and anti‐inflammation.^20^ Decreased molecular weight of HA has been found in the synovial fluid of patients with temporomandibular joint (TMJ) disorders.^21^ Studies have shown a significant correlation between the molecular weight of HA and its biological effects.^22^, ^23^ LMW‐HA promotes M1 macrophage polarization,^24^ while HMW‐HA attenuates synovitis by promoting M2 macrophage polarization.^25^ It has been demonstrated that HMW‐HA is effective in the treatment of TMJ inflammation in animal experiments^26^ and clinical.^27^ However, the relationship of HA, ALPK1 and macrophage polarization in TMJ synovitis remains unclear.

In the present study, the alteration of ALPK1 in the inflamed synovium and synovial fluid of human TMJs was analysed. Subsequently, a CFA‐induced TMJ synovitis model and ALPK1 knockout mice were used to investigate the function of ALPK1 in synovial inflammation as well as M1 macrophage polarization. Moreover, the role of ALPK1 and HA in M1 macrophage polarization was explored with the RAW264.7 cell line. Our results suggested that ALPK1 played an important role in the pathogenesis of TMJ synovitis and provided valuable insights for the treatment of TMJOA with HMW‐HA.

## 2 MATERIALS AND METHODS

### 2.1 Clinical samples

Synovial fluid samples were collected from 11 patients with clinically confirmed TMJ synovitis and 8 patients suffered from TMJ anterior disc displacement without synovitis. Specimens of inflamed synovium were obtained from five patients with TMJOA who were observed with obvious TMJ synovitis under direct vision in arthroplasty. The control synovial tissues were derived from five patients with condylar hyperplasia. All of the experiment patients were on the premise of informed consent. The ethics approval was granted by the Ethics Committee of Wuhan University Hospital of Stomatology (approval No. 2019‐A15). All methods were carried out in accordance with relevant guidelines and regulations.

### 2.2 Animal model

Eight‐week‐old C57/BL6 male wild‐type (WT) mice and ALPK1^−/−^ mice were employed in this experiment. ALPK1 knockout mice were produced as previously described.^28^ Synovial inflammation was induced by bilateral injection of 20 μL CFA (F5881; Sigma) into the anterosuperior compartment of TMJ, and the control group received isopycnic saline. Specimens from WT and ALPK1^−/−^ mice were sacrificed 1 or 2 weeks after CFA injection. All animal studies were approved by the Ethics Committee for Animal Research, School and Hospital of Stomatology, Wuhan University (approval No. S07919110A), were operated in accordance with national guidelines for the housing and care of laboratory animals, and conformed to the ARRIVE 2.0 guidelines.

### 2.3 Histologic staining

TMJ samples were collected, using 4% paraformaldehyde and 10% EDTA for fixation and decalcification, and following embedded in paraffin, continuous mid‐sagittal sections of 4 μm were cut for subsequent staining. After dewaxing and gradient hydration, haematoxylin and eosin (HE) staining was performed to quantify the synovial inflammation.

Immunofluorescence staining was incubated with the designated primary antibody overnight at 4°C and then conjugated the primary antibody with a fluorescent secondary antibody to visualize staining, and 4′,6‐diamidino‐2‐phenylindole (DAPI) reagent was used to develop nuclei. Primary antibodies were as follows: rabbit anti‐ALPK1 (1:300, 19107‐1‐AP; Proteintech), mouse anti‐CD68 (1:500, 66,231‐2‐Ig, Proteintech), rabbit anti‐Vimentin (1:200, 10366‐1‐AP, Proteintech), rabbit anti F4/80 (1:200, GB11027, Servicebio) and rabbit anti‐INOS (1:500, GB11119, Servicebio).

Immunohistochemical staining was to incubate with the designated primary antibody at 4°C overnight, then with the designated secondary antibody for 30 min, and finally developed the colour with DAB (DAB‐0031, Bio technologies), followed by counterstaining of nuclei with haematoxylin. Primary antibodies were as follows: rabbit anti‐TNF‐α (1:400, 11948S, Cell Signaling Technology), rabbit anti‐IL‐1β (1:400, YT2322, Immunoway) and rabbit anti‐IL‐6 (1:400, A14687, ABclonal).

### 2.4 Cell culture

RAW264.7 (provided by doc. Huang Xin) was cultured in Dulbecco's Modified Eagle Medium (DMEM, SH30022.01, Hyclone) containing 10% fetal bovine serum (FBS) and 1% penicillin–streptomycin in a 37°C incubator. Subsequently, the cells were induced with 100 ng/mL lipopolysaccharide (LPS, Sigma, L2654, USA) and 20 ng/mL interferon‐gamma (IFN‐γ, Novoprotein, CM41, China) for 24 h in a fresh medium to stimulate M1 polarization. In the presence of LPS and IFN‐γ, 20 ng/mL rhALPK1 (H00080216‐Q01; Abnova) was added to examine the effect of rhALPK1 on macrophage polarization. 2 mg/mL LMW‐HA (MACKLIN, H874944‐1g, China, molecular weight 10–100 KDa) or 2 mg/mL HMW‐HA (MACKLIN, H909939‐5g, China, molecular weight 1500–2500 KDa) was added to examine the effect of HA on macrophage polarization.

### 2.5 Cell viability assay with CCK‐8

Cells were seeded in 96‐well plates at a density of 5 × 10^4^ per well. Then, the cells were treated with rhALPK1 at concentrations of 0, 2, 5, 10, 15, 20 and 25 ng/mL, and the cells were stored in a 37°C, 5% CO_2_ incubator. After 24 h, 200 μL of 10% CCK‐8 solution diluted in DMEM medium was add to each well. After further incubation for 2 h, use a microplate reader to measure the absorbance of each well at a wavelength of 450 nm.

### 2.6 Enzyme‐linked immunosorbent assay (ELISA)

The synovial fluid and cell supernatant were tested according to the protocols of the ELISA kit (Human ALPK1 ELISA Kit, JL14242, Jianglai Biotechnology; Mouse IL‐1β ELISA Kit, CME0006, Sizhengbai Biotechnology; Mouse TNF‐α ELISA Kit, CME0004, Sizhengbai Biotechnology). In addition, the relative concentration of ALPK1 in synovial fluid is calibrated using the total protein concentration measured by BCA.

### 2.7 Western blot

Cells were first lysed with RIPA lysis buffer to collect cellular proteins. Then, the protein was separated by 10% SDS‐PAGE and then electro‐transferred to PVDF membrane. The protein on the membrane was detected with a suitable primary antibody. Finally, the protein was incubated with the secondary antibody for 1 h, and the protein signal was detected by the luminometer ECL. Primary antibodies were as follows: rabbit anti‐INOS (1:1000, GTX130246, GeneTex), mouse anti‐β‐actin (1:5000, RAB0100, Frdbio), rabbit anti‐IL‐1β (1:1000, YT2322, Immunoway), mouse anti‐PKM2 (1:1000, 60268‐1‐Ig, Proteintech), Mouse anti‐Histone H3.1(1:1000, GB11102, Servicebio), rabbit anti‐TNF‐α (1:1500, GTX110520, GeneTex), rabbit anti‐ALPK1 (1:6000, Abcam) and mouse anti‐GAPDH (1:5000, RAB0101, Frdbio).

### 2.8 Quantitative real‐time polymerase chain reaction (qPCR)

Total RNA was extracted with Trizol, and then, 1ug RNA was reversely transcribed to cDNA according to the manufacturer's protocol (Takara). The qRT‐PCR reactions were performed on the Biorad CFXTM Connect Real‐Time System according to the manufacturer's protocol (Takara), as described previously.^29^ Primer sequences were as follows: CD86 (forward: ACTGTCAGTGATCGCCAACT, reverse: TAGGTTTCGGGTGACCTTGC); INOS (forward: GGAGCATCCCAAGTACGAGT, reverse: CCAATCTCGGTGCCCATGTA); TNF‐α (forward: AGGCACTCCCCCAAAAGATG, reverse: CCATTTGGGAACTTCTCATCCC); IL‐1β (forward: AGGAGAACCAAGCAACGACA, reverse: CTTGGGATCCACACTCTCCAG); ALPK1 (forward: GTGGCAATACAAACAAGCCAT, reverse: CGGTCCATCAAGAAGACAATAG); IL‐6 (forward: ACAAGTCCGGAGAGGAGACT, reverse: AATTGCCATTGCACAACTCTT); β‐actin (forward: CGCAGCCACTGTCGAGTC, reverse: GTCATCCATGGCGAACTGGT). The housekeeping gene β‐actin was used as an internal reference.

### 2.9 Statistical analysis

Statistical analyses were conducted in Prism 8.0 (GraphPad prism8), and *p* < 0.05 was considered statistically significant. Comparisons between two groups were performed with Student's *t*‐test. Comparisons between three and six groups were performed with one‐way analysis of variance. Notably, human data were considered nonparametric distributions and comparisons between two independent groups were analysed by Mann–Whitney *U*‐test.

## 3 RESULTS

### 3.1 ALPK1 is highly expressed in macrophages of TMJ inflamed synovial fluid and synovium

**FIGURE 1:**
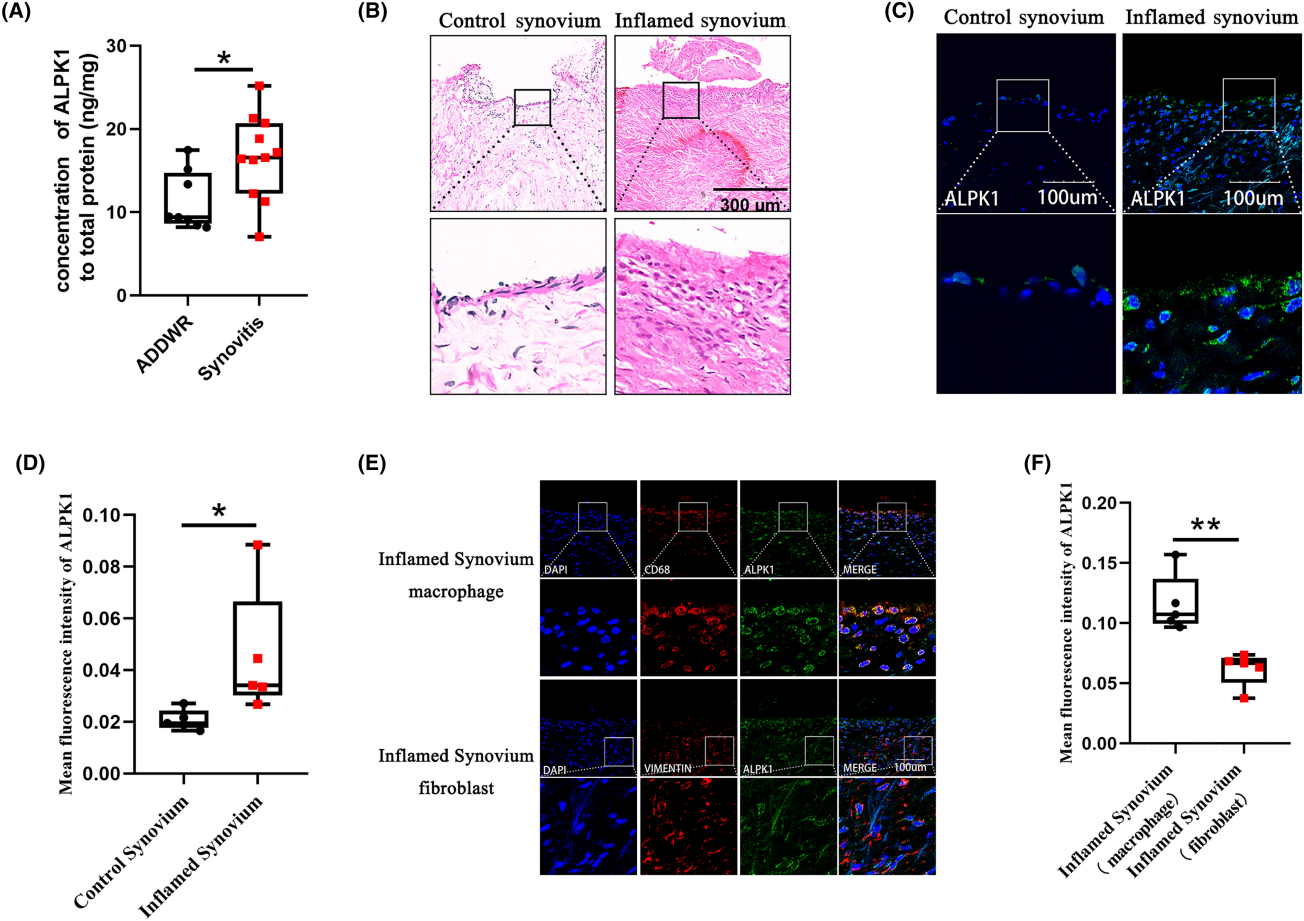
ALPK1 is highly expressed in macrophages of TMJ inflamed synovium. (A) Upregulation of ALPK1 in the synovial fluid of patients with TMJ synovitis was discovered by ELISA, as compared with patients with anterior disc displacement with reduction (ADDWR). Mann–Whitney *U*‐test, minimum to maximum with all points, *n* = 8–11, *p* = 0.0328. (B) Haematoxylin and eosin (H&E) staining showed the control synovium and inflamed synovial. (C) The expression of ALPK1 was examined in TMJ synovium. (D) Quantitative analysis of ALPK1 in TMJ synovium. Mann–Whitney *U*‐test, minimum to maximum with all points, *n* = 5, *p* = 0.0159. (E) The expression of ALPK1 was examined in TMJ synovial macrophages and TMJ synovial fibroblasts. (F) Quantitative analysis of ALPK1 in TMJ synovial macrophages and TMJ synovial fibroblasts. Mann–Whitney *U*‐test, minimum to maximum with all points, *n* = 5, *p* = 0.0079. *p < 0.05. **p < 0.01.

First, upregulation of ALPK1 was discovered in synovial fluid from patients with TMJ synovitis in comparison with patients with TMJ anterior disc displacement by ELISA analysis (Figure 1A). Figure 1B showed the control synovium and inflamed synovium of TMJ by HE staining. The immunofluorescence analysis showed that the expression of ALPK1 in inflamed synovium was higher than that in control synovium (Figure 1C,D). In addition, synovial macrophages in the inflamed synovium showed abundant expression of ALPK1, whereas expression by synovial fibroblast was barely detectable (Figure 1E,F). These results suggested that a high level of ALPK1 was discovered in macrophages of inflamed synovium and may be involved in TMJ synovitis.

### 3.2 LMW‐HA enhances the expression of ALPK1 and M1 macrophage polarization

**FIGURE 2:**
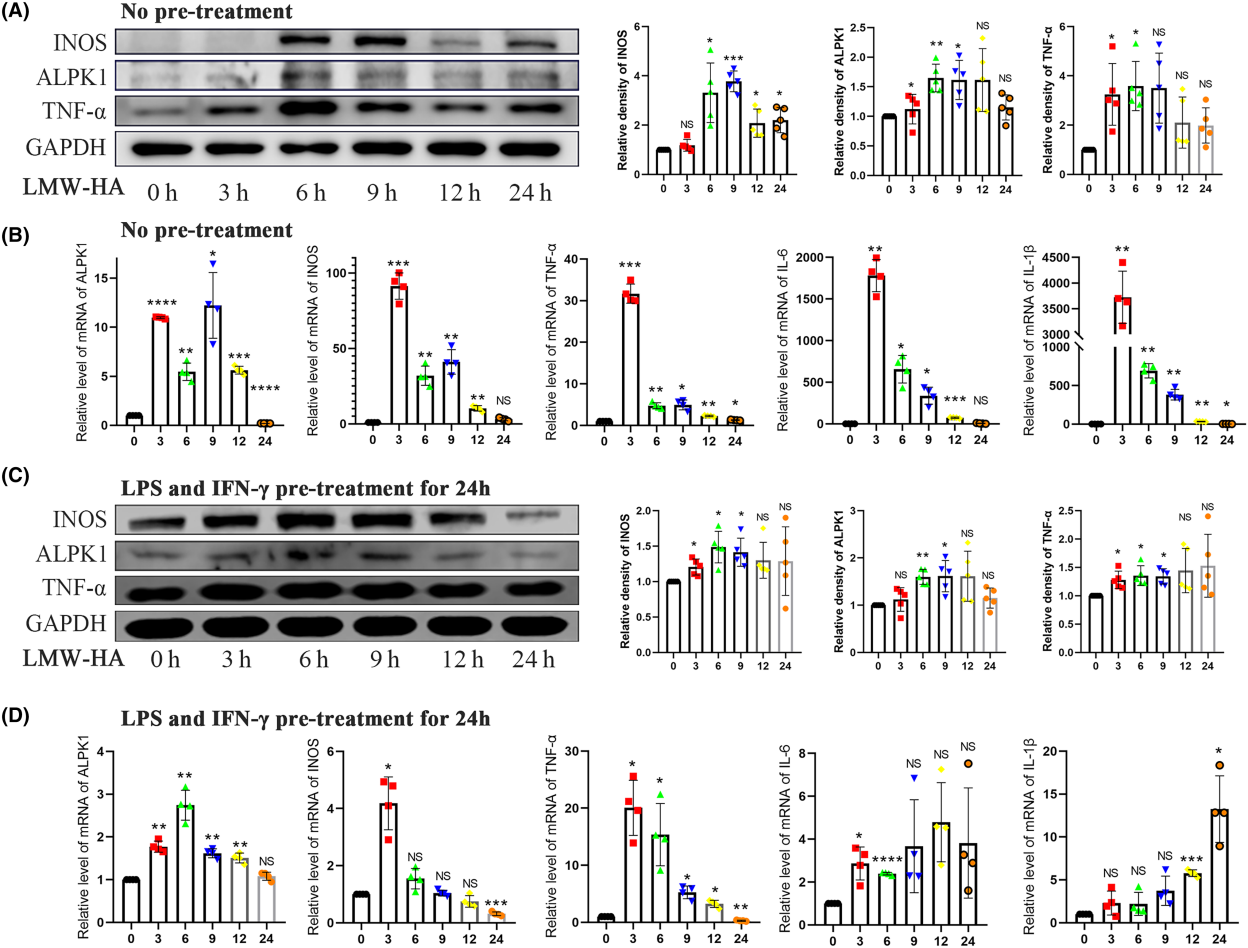
LMW‐HA enhances the expression of ALPK1 and M1 macrophage polarization. (A) Effect of LMW‐HA on the expression of ALPK1, INOS, TNF‐α in RAW264.7 cells by western blot. One‐way analysis of variance, mean ± SEM, *n* = 5. (B) Effect of LMW‐HA on the expression of ALPK1, INOS, TNF‐α, IL‐6 and IL‐1β in RAW264.7 cells by qPCR. One‐way analysis of variance, mean ± SEM, *n* = 4. (C) Effect of LMW‐HA on the expression of ALPK1, INOS and TNF‐α in RAW264.7 cells pretreated with LPS (100 ng/mL) and IFN‐γ (20 ng/mL) for 24 h by western blot. One‐way analysis of variance, mean ± SEM, *n* = 5. (D) Effect of LMW‐HA on the expression of ALPK1, INOS, TNF‐α, IL‐6 and IL‐1β in RAW264.7 cells pretreated with LPS (100 ng/mL) and IFN‐γ (20 ng/mL) for 24 h by qPCR. One‐way analysis of variance, mean ± SEM, *n* = 4. *p < 0.05. **p < 0.01. ***p < 0.001. ****p < 0.0001. NS, no significance.

Reduced molecular weight of HA has been found in the pathogenesis of TMJOA.^21^ So, it aroused our interest that what is the effect of LMW‐HA on ALPK1, a possible synovitis‐associated molecule in macrophages. In our results, western blot analysis revealed that ALPK1, as well as the inflammatory factors INOS and TNF‐α, are elevated under stimulation by LMW‐HA (Figure 2A,C). qPCR assay showed that the expression of ALPK1 and M1 macrophage‐associated genes INOS, TNF‐ α, IL‐6 and IL‐1β was increased under treatment with LMW‐HA (Figure 2B,D). Our study showed that the pro‐inflammatory substance LMW‐HA promoted the expression of ALPK1, further suggesting that ALPK1 may act as a pro‐inflammatory gene in TMJ synovitis.

### 3.3 ALPK1 promotes M1 macrophage polarization in vitro

**FIGURE 3:**
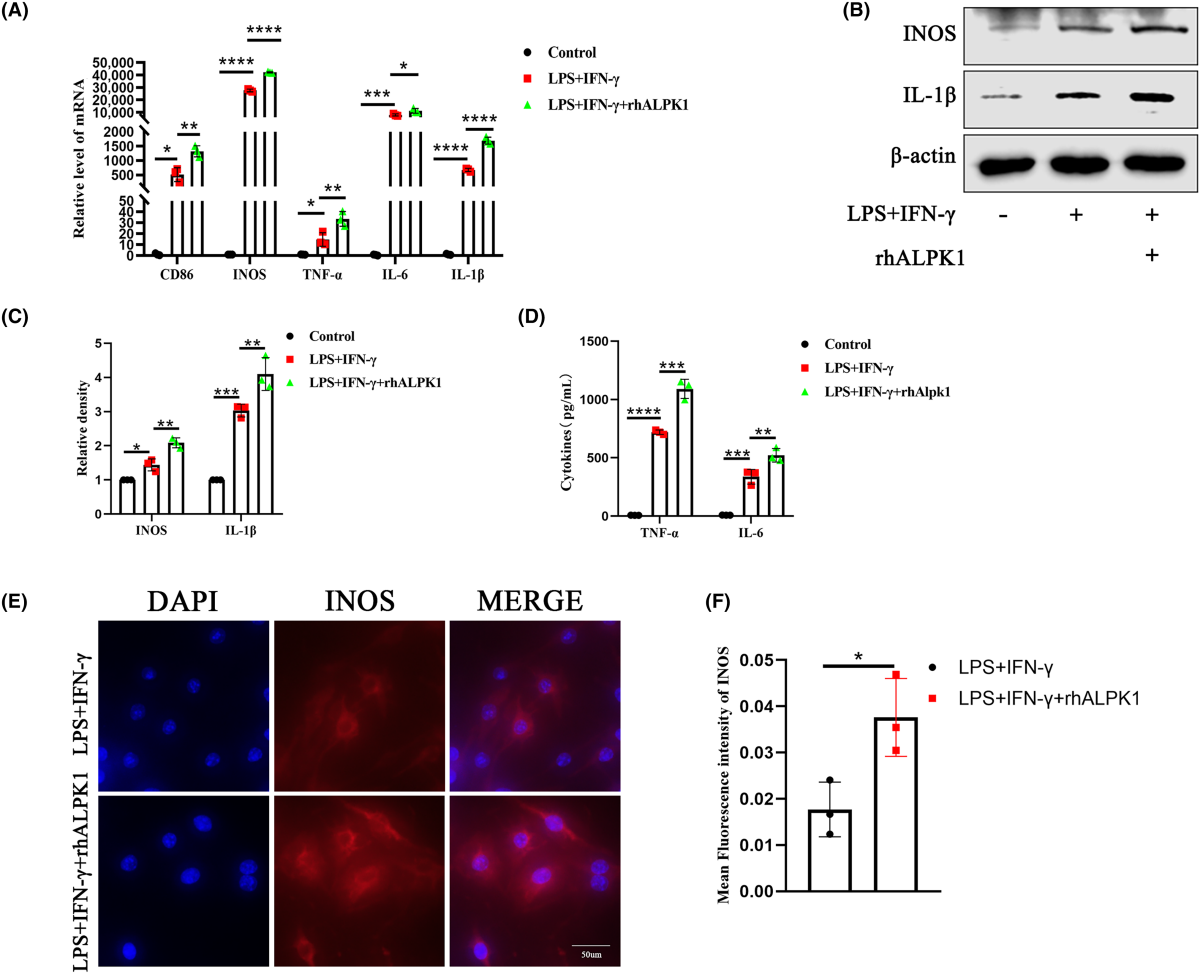
ALPK1 promotes M1 macrophage polarization in vitro. (A) qPCR analysis exhibited that rhALPK1 (20 ng/mL) increased the production of CD86, INOS, TNF‐α, IL‐6 and IL‐1β in RAW264.7cells. Relative levels of CD86: Control group versus LPS + IFN‐γ group, *p* = 0.0224. LPS + IFN‐γ group versus LPS + IFN‐γ + rhALPK1 group, *p* = 0.0026. Relative levels of INOS: Control group versus LPS + IFN‐γ group, *p* < 0.0001. LPS + IFN‐γ group versus LPS + IFN‐γ + rhALPK1 group, *p* < 0.0001. Relative levels of TNF‐α: Control group versus LPS + IFN‐γ group, *p* = 0.0342. LPS + IFN‐γ group versus LPS + IFN‐γ + rhALPK1 group, *p* = 0.0087. Relative levels of IL‐6: Control group versus LPS + IFN‐γ group, *p* = 0.0003. LPS + IFN‐γ group versus LPS + IFN‐γ + rhALPK1 group, *p* = 0.0252. Relative levels of IL‐1β: Control group versus LPS + IFN‐γ group, *p* < 0.0001. LPS + IFN‐γ group versus LPS + IFN‐γ + rhALPK1 group, *p* < 0.0001. One‐way analysis of variance, mean ± SEM, *n* = 3. (B) rhALPK1(20 ng/mL) increased protein expression of INOS and IL‐1β in LPS + IFN‐γ‐treated RAW264.7 cells. (C) Quantitative analysis of the relative density of INOS and IL‐1β. Relative density of INOS: Control group versus LPS + IFN‐γ group, *p* = 0.0115. LPS + IFN‐γ group versus LPS + IFN‐γ + rhALPK1 group, *p* = 0.0017. Relative density of IL‐1β: Control group versus LPS + IFN‐γ group, *p* = 0.0004. LPS + IFN‐γ group versus LPS + IFN‐γ + rhALPK1 group, *p* = 0.0091. One‐way analysis of variance, mean ± SEM, *n* = 3. (D) ELISA analysis exhibited that rhALPK1 (20 ng/mL) increased the secretion of TNF‐α and IL‐6 in RAW264.7cells. Cytokines of TNF‐α: Control group versus LPS + IFN‐γ group, *p* < 0.0001. LPS + IFN‐γ group versus LPS + IFN‐γ + rhALPK1 group, *p* = 0.0002. Cytokines of IL‐6: Control group versus LPS + IFN‐γ group, *p* = 0.0004. LPS + IFN‐γ group versus LPS + IFN‐γ + rhALPK1 group, *p* = 0.0071. One‐way analysis of variance, mean ± SEM, *n* = 3. (E) ALPK1 increases the expression of INOS by Immunofluorescence staining. (F) Quantitative analysis of the relative density of INOS. Student's *t*‐test, *n* = 3. *p < .05.**p < .01.***p < 0.001.****p < 0.0001.

To study the role of ALPK1 in macrophages, we determined the optimal concentration of rhALPK1 in RAW264.7 cells. As shown in Figure S1, when RAW264.7 cells were treated with different rhALPK1 concentrations of 2, 5, 10, 15, 20 and 25 ng/mL, the cell viability was almost 100%, and at the concentration of 20 ng/mL, the viability of RAW264.7 cells is closest to 100%. Based on the preliminary results, the experimental concentration of rhALPK1 was finally determined to be 20 ng/mL. As a possible pro‐inflammatory molecule, does ALPK1 promote M1 macrophage in vitro? In our study, we used rhALPK1 (20 ng/mL) to stimulate RAW264.7 cells in the presence of LPS and IFN‐γ. The expression of INOS, CD86, TNF‐α, IL‐6 and IL‐1β was elevated in RAW264.7 cells by qPCR analysis (Figure 3A). Consistently, protein analysis results showed that ALPK1 could promote the expression of INOS and IL‐1β (Figure 3B,C). Meanwhile, ALPK1 promoted the secretion of inflammatory factors including TNF‐α and IL‐6 (Figure 3D). Consistently, immunofluorescence showed that ALPK1 promoted the expression of INOS (Figure 3E,F). Taken together, these findings suggested that ALPK1 promoted M1 macrophage polarization in RAW264.7 cells.

### 3.4 ALPK1 deficiency alleviates CFA‐induced synovial inflammation in TMJ of mice

**FIGURE 4:**
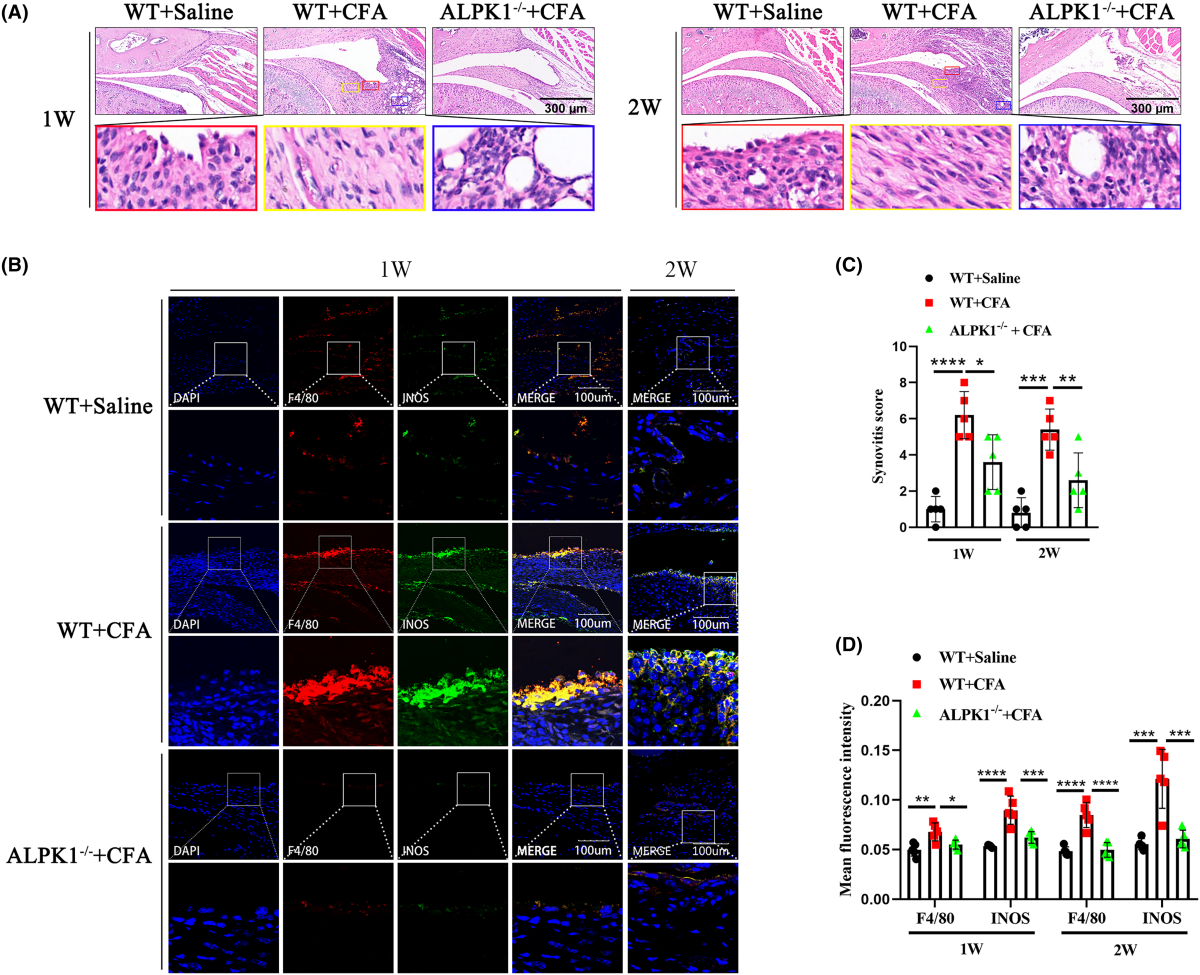
ALPK1 deficiency alleviates CFA‐induced synovial inflammation in TMJ of mice. (A) ALPK1 deficiency alleviated CFA‐induced synovitis of inflamed TMJs in the first and second weeks, with the phenomena of decreased synovial cell layers (red solid box), stromal cell density (yellow solid box) and inflammatory cell infiltration (blue solid box). (B) The expression of F4/80 (macrophage marker) and INOS (M1‐like macrophage marker) was examined in the TMJ synovium of mice by Immunofluorescence. (C) Synovitis score of mice. 1 week: Comparing WT + Saline group versus WT + CFA group, *p* < 0.0001. Comparing WT + CFA group versus ALPK1^−/−^ + CFA group, *p* = 0.0106. 2 weeks: Comparing WT + Saline group versus WT + CFA group, *p* = 0.0001. Comparing WT + CFA group versus ALPK1^−/−^ + CFA group, *p* = 0.0057. (D) Quantitative analysis of the fluorescence intensity of F4/80 and INOS in TMJ synovium of mice. Fluorescence intensity of F4/80 for 1 week: Comparing WT + Saline group versus WT + CFA group, *p* = 0.0029. Comparing WT + CFA group versus ALPK1^−/−^ + CFA group, *p* = 0.0235. Fluorescence intensity of INOS for 1 week: Comparing WT + Saline group versus WT + CFA group, *p* < 0.0001. Comparing WT + CFA group versus ALPK1^−/−^ + CFA group, *p* = 0.0008. Fluorescence intensity of F4/80 for 2 weeks: Comparing WT + Saline group versus WT + CFA group, *p* < 0.0001. Comparing WT + CFA group versus ALPK1^−/−^ + CFA group, *p* < 0.0001. Fluorescence intensity of INOS for 2 weeks: Comparing WT + Saline group versus WT + CFA group, *p* = 0.0002. Comparing WT + CFA group versus ALPK1^−/−^ + CFA group, *p* = 0.0004. One‐way analysis of variance, mean ± SEM, *n* = 5. *p < 0.05.**p < 0.01. ***p<0.001. ****p < 0.0001.

**FIGURE 5:**
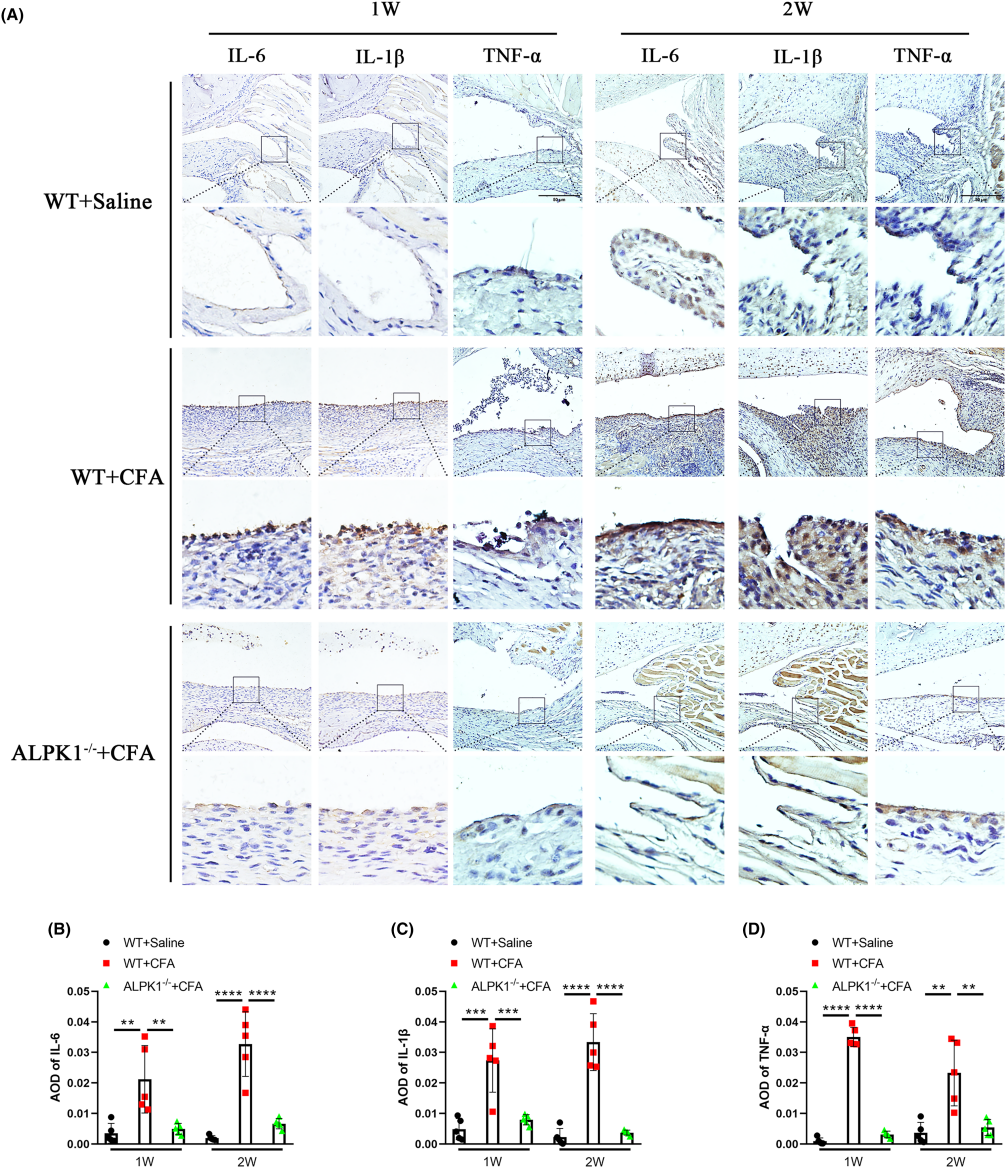
ALPK1 deficiency reduces the expression of pro‐inflammatory factors in synovial macrophages in TMJ of mice. (A) ALPK1 deficiency reduces the expression of IL‐6, IL‐1β and TNF‐α in the synovium of TMJ. (B) Average optical density (AOD) of positive staining for IL‐6 in TMJ synovium of mice. 1 week: Comparing WT + Saline group versus WT + CFA group, *p* = 0.0025. Comparing WT + CFA group versus ALPK1^−/−^ + CFA group, *p* = 0.0047. 2 weeks: Comparing WT + Saline group versus WT + CFA group, *p* < 0.0001. Comparing WT + CFA group versus ALPK1^−/−^ + CFA group, *p* < 0.0001. (C) Average optical density (AOD) of positive staining for IL‐1β in TMJ synovium of mice. 1 week: Comparing WT + Saline group versus WT + CFA group, *p* = 0.0002. Comparing WT + CFA group versus ALPK1^−/−^ + CFA group, *p* = 0.0008. 2 weeks: Comparing WT + Saline group versus WT + CFA group, *p* < 0.0001. Comparing WT + CFA group versus ALPK1^−/−^ + CFA group, *p* < 0.0001. (D) Average optical density (AOD) of positive TNF‐α staining in TMJ synovium of mice. 1 week: Comparing WT + Saline group versus WT + CFA group, *p* < 0.0001. Comparing WT + CFA group versus ALPK1^−/−^ + CFA group, *p* < 0.0001. 2 weeks: Comparing WT + Saline group versus WT + CFA group, *p* = 0.0011. Comparing WT + CFA group versus ALPK1^−/−^ + CFA group, *p* = 0.0022. One‐way analysis of variance, mean ± SEM, *n* = 5. **p < 0.01. ***p < 0.001. ****p < 0.0001.

To further investigate the role of ALPK1 in TMJ synovitis, we established an ALPK1 knockout mice. In the current study, TMJ synovitis mice were employed and harvested 1 and 2 weeks after CFA injection. In contrast to WT mice, ALPK1^−/−^ mice exhibited decreased synovial cell layers, stromal cell density and inflammatory cell infiltration by HE staining (Figure 4A), as well as significantly lower synovitis scores (Figure 4C). We further investigated the role of ALPK1 in synovial macrophages in TMJ inflammation. Compared with WT mice, a marked decreased level of F4/80 (a macrophage marker) was detected in ALPK1^−/−^ mice, together with a significantly reduced level of INOS (a M1‐like macrophage marker) predominantly in the intimal lining layer (Figure 4B,D). In addition, in contrast to WT mice, ALPK1−/− mice exhibited a significantly reduced level of IL‐6, IL‐1β and TNF‐α in synovium (Figure 5A,B). These results indicated that ALPK1 could promote TMJ synovitis and M1 macrophage polarization.

### 3.5 ALPK1 promotes M1 macrophage polarization by increasing the nuclear translocation of PKM2

**FIGURE 6:**
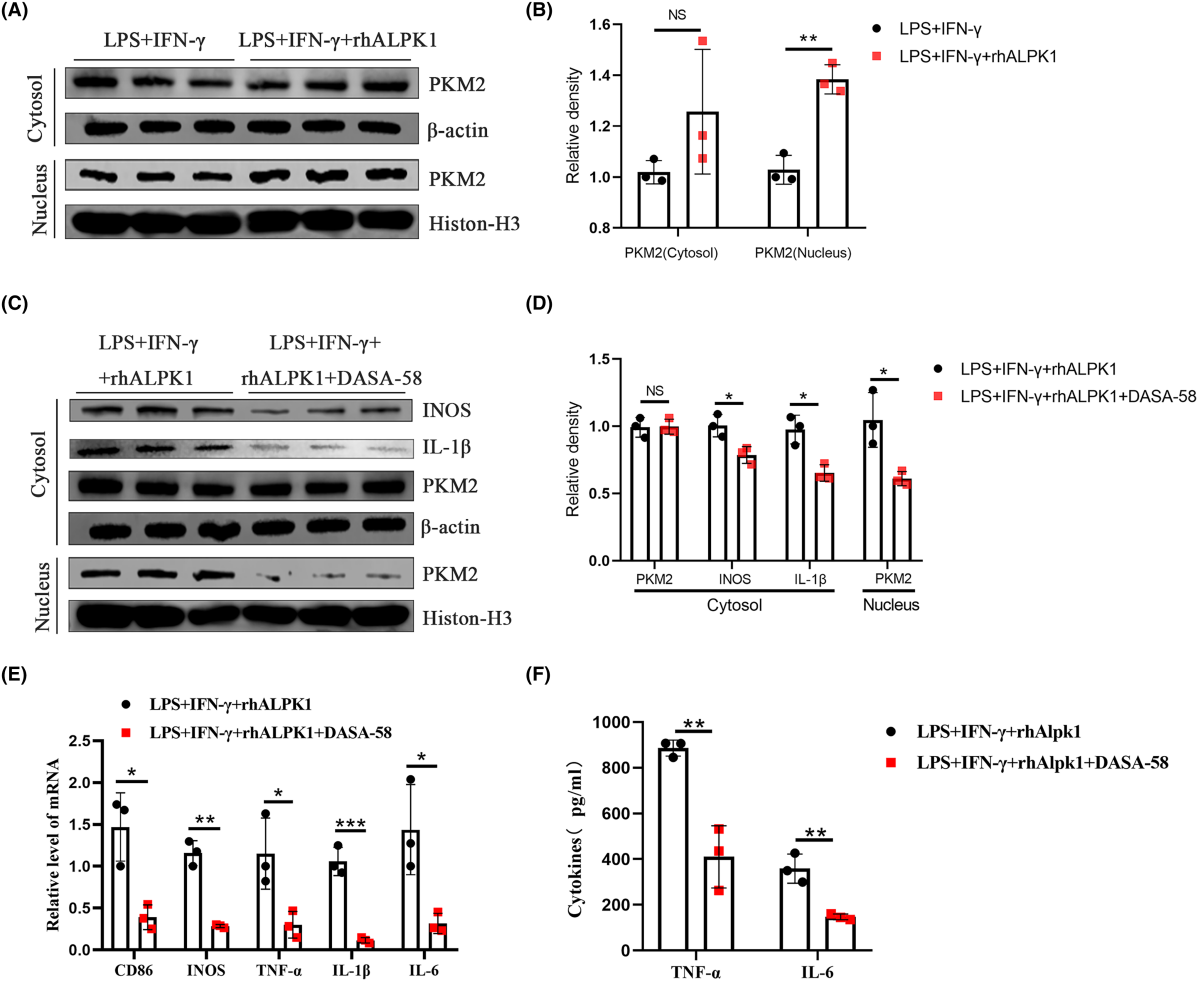
ALPK1 promotes M1 macrophage polarization by increasing the nuclear translocation of PKM2. (A) RAW264.7 cells were treated with rhALPK1 (20 ng/mL) in the presence of LPS and IFN‐γ for 24 h. Cytosolic and nuclear protein fractions were immunoblotted with the indicated antibodies. (B) Quantitative analysis of the relative density of PKM2. PKM2 (Cytosol), *p* = 0.8088. PKM2(Nucleus), *p* = 0.0016. (C) RAW264.7 cells were treated with 50 umol/L DASA‐58(B6025, APExBIO) in the presence of LPS, IFN‐γ and rhALPK1 for 24 h. Cytosolic and nuclear protein fractions were immunoblotted with the indicated antibodies. (D) Quantitative analysis of the relative density of INOS, IL‐1β and PKM2. Relative density of INOS in Cytosol, *p* = 0.0221. Relative density of IL‐1β in Cytosol, *p* = 0.0105. Relative density of PKM2 in Cytosol, *p* = 0.9317. Relative density of PKM2 in Nucleus, *p* = 0.0226. (E) QPCR analysis showed that 50 umol/L DASA‐58 inhibits the expression of CD86, INOS, TNF‐α, IL‐1β and IL‐6 in RAW264.7 cells. CD86, *p* = 0.0126. INOS, *p* = 0.0079. TNF‐α, *p* = 0.0315. IL‐1β, *p* = 0.0007. IL‐6, *p* = 0.0244. (F) DASA‐58 inhibits the secretion of TNF‐α and IL‐6 in RAW264.7 cells. TNF‐α, *p* = 0.0048. IL‐6, *p* = 0.0042. Student's *t*‐test, mean ± SEM, *n* = 3. *p < 0.05. **p < 0.01. ***p < 0.001. NS, No Significance.

Numerous studies have shown that nuclear PKM2 plays an important role in M1 macrophage polarization.^30^, ^31^ However, whether PKM2 mediates ALPK1‐promoted M1 macrophage polarization remains to be explored. In our study, as shown in Figure 6A,B, PKM2 nuclear translocation was increased, but PKM2 in cytosolic had no significant change in RAW264.7 cells stimulated by rhALPK1. To further explore the role of PKM2 in ALPK1 promoting M1 macrophage polarization, DASA‐58, which is a specific potent activator of PKM2 and can prevent PKM2 nuclear translocation,^30^, ^31^ was used. The results showed that DASA‐58 significantly reversed the nuclear translation of PKM2 and the expression of INOS and IL‐1β promoted by ALPK1 (Figure 6C,D). Consistently, the expression of INOS, CD86, TNF‐α, IL‐6 and IL‐1β was reversed by DASA‐58 (Figure 6E). Similarly, the secretion of TNF‐α and IL‐6 was reversed by DASA‐58 (Figure 6F). Collectively, these findings demonstrated that ALPK1 promoted M1 macrophage polarization by increasing nuclear translocation of PKM2.

### 3.6 HMW‐HA inhibits the expression of ALPK1 and M1 macrophage polarization

**FIGURE 7:**
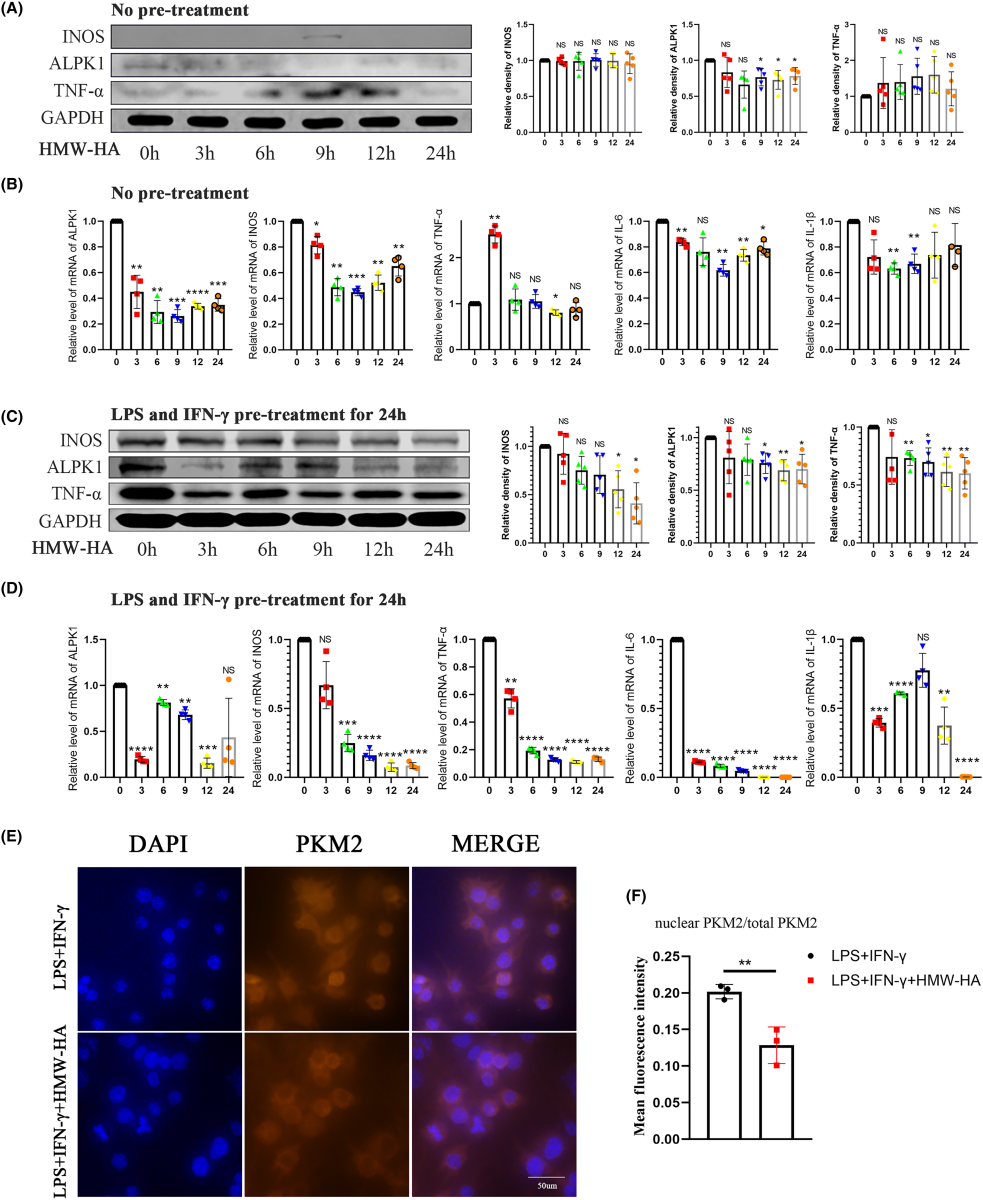
HMW‐HA inhibits the expression of ALPK1 and M1 macrophage polarization. (A) Effect of HMW‐HA on the expression of ALPK1, INOS, TNF‐α in RAW264.7 cells by western blot. One‐way analysis of variance, mean ± SEM, *n* = 5. (B) Effect of HMW‐HA on the expression of ALPK1, INOS, TNF‐α, IL‐6 and IL‐1β in RAW264.7 cells by qPCR. One‐way analysis of variance, mean ± SEM, *n* = 4. (C) Effect of HMW‐HA on the expression of ALPK1, INOS and TNF‐α in RAW264.7 cells pretreated with LPS (100 ng/mL) and IFN‐γ (20 ng/mL) for 24 h by western blot. One‐way analysis of variance, mean ± SEM, *n* = 5. (D) Effect of HMW‐HA on the expression of ALPK1, INOS, TNF‐α, IL‐6 and IL‐1β in RAW264.7 cells pretreated with LPS (100 ng/mL) and IFN‐γ (20 ng/mL) for 24 h by qPCR. One‐way analysis of variance, mean ± SEM, *n* = 4. (E) HMW‐HA inhibited the nuclear translocation of PKM2 in the absence of LPS and IFN‐γ pretreatment. (F) Quantitative analysis of the relative density of PKM2. Student's *t*‐test, *n* = 3. *p < 0.05. **p < 0.01. ***p < 0.001. ****p < 0.0001. NS, no significance.

HMW‐HA is often used for the treatment of TMJ inflammation.^27^ The impact of HMW‐HA on the expression of ALPK1 and the activation of macrophages has attracted our attention. In this study, RAW264.7 cells were treated by HMW‐HA with or without LPS and IFN‐γ pretreatment. Western blot analyses showed that HMW‐HA inhibited the expression of ALPK1 (Figure 7A,C). But changes in the expression of INOS and TNF‐α were not significant under HMW‐HA stimulation in the absence of LPS and IFN‐γ pretreatment (Figure 7A). Meanwhile, qPCR experiments showed that HMW‐HA decreased the expression of ALPK1, INOS, TNF‐α, IL‐6 and IL‐1β (Figure 7B,D). In addition, immunofluorescence showed that HMW‐HA inhibited the nuclear translocation of PKM2 in the absence of LPS and IFN‐γ pretreatment (Figure 7E,F). Taken together, these findings suggested that HMW‐HA had an anti‐inflammatory effect and inhibited the expression of ALPK1 and M1 macrophage polarization.

## 4 DISCUSSION

Recent studies have demonstrated that ALPK1 is expressed in the testes of mice,^32^ kidneys of humans^33^ and intestinal mucosa of humans.^34^ In the present study, it was found that ALPK1 was highly expressed in the synovial fluid and inflamed synovium of TMJ patients. Macrophages of inflamed synovium showed abundant expression of ALPK1, whereas expression by synovial fibroblasts was barely detectable. The possible reason is that certain factors are highly expressed in macrophages in the specific environment. Similarly, Kong, Q. et al. found that expression of HSPA12A in liver macrophages was higher than that in Hepatocytes.^35^ Intriguingly, although ALPK1 was preferably expressed in synovial macrophages, there is some positive staining of ALPK1 in a few synovial fibroblasts, which may be due to their cluster heterogeneity.^36^

At present, studies have confirmed that ALPK1 is a promoting factor in several inflammation‐related diseases.^11^, ^12^ Meanwhile, M1 macrophage polarization plays an important role in the development of joint inflammation.^37^ Moreover, M1 macrophages play a more critical role in cartilage damage in TMJOA patients than other common cell populations.^38^ Inhibition of M1 macrophage polarization alleviates TMJOA.^39^ The molecular weight of HA decreases in TMJOA,^21^ and many studies have suggested that LMW‐HA promoted M1 macrophage polarization.^40^, ^41^ In our experiments, we found that LMW‐HA not only promoted M1 macrophage polarization but also the expression of ALPK1. Furthermore, rhALPK1 promoted the expression of M1 macrophage markers CD86 and INOS, and the expression of inflammatory cytokines TNF‐α, IL‐1β and IL‐6 in RAW264.7 cells. ALPK1 knockout mice and CFA‐induced TMJ synovitis model were used for further studies. Our study found that ALPK1 knockout mice exhibited decreased synovial cell layers, stromal cell density and inflammatory cell infiltration, as well as significantly lower synovitis scores, indicating that ALPK1 could promote the development of TMJ synovitis. These results suggested that ALPK1 aggravated synovitis by promoting M1 macrophage polarization.

Glycolysis plays an important role in M1 macrophage polarization.^42^ In M1 macrophages, there is a Warburg effect similar to that observed in tumour cells.^43^ The Warburg effect means that in the presence of a lot of oxygen, glucose is still converted into lactate, that is, cells produce energy through glycolysis.^44^ As a key rate‐limiting enzyme for glycolysis, PKM2 has been shown an important role in the M1 macrophage polarization.^18^ In this study, we demonstrated that ALPK1 could promote nuclear translocation of PKM2 in macrophages. Importantly, DASA‐58 significantly reversed the enhanced production of INOS, CD86 and IL‐1β, and secretion of IL‐6 and TNF‐α in these cells induced by rhALPK1, demonstrating that ALPK1 regulates synovitis by promoting nuclear PKM2‐mediated M1 macrophage polarization.

As a normal component of the TMJ synovial fluid, HMW‐HA is widely used clinically in the treatment of TMJ inflammation.^27^ In recent years, HA‐related biological materials have also shown good therapeutic effects in TMJ inflammation.^45^ In addition to this, HA‐derived biocompatible materials have also been used to repair perforated TMJ discs.^46^ Veronica Iturriaga et al. found that compared to HMW‐HA, LMW‐HA had better therapeutic effects on MIA‐induced TMJOA in rabbits.^47^ However, the experimental results of Wen, S. et al. showed that the therapeutic effect of HMW‐HA is better than LMW‐HA.^48^ In addition, one study found that LMW‐HA reduced survival in mice with post‐intensive care syndrome in the short term but increased survival in the long term.^49^ In vitro studies, we found that LMW‐HA promoted M1 macrophage polarization and the expression of ALPK1. Besides, HMW‐HA had an inhibitory effect on inflammation. We consider that HMW‐HA inhibits inflammation and the clinical therapeutic effects of HMW‐HA are associated with inhibition of the expression of ALPK1 and M1 macrophage polarization, while LMW‐HA cannot be simply defined as a pro‐inflammatory factor. HA mainly functions through CD44, TLR2 and TLR4.^50^ Specifically, LMW‐HA promotes inflammation by activating the TLR2 and TLR4 signalling pathways,^51^ while its binding affinity with CD44 is lower.^52^ HMW‐HA inhibits inflammation by regulating the TLR2, TLR4 and CD44 signalling pathways.^53^, ^54^ HA may have different effects due to differences in molecular weight and microenvironment.

In summary, our results provide the initial understanding that ALPK1 regulates synovitis by promoting nuclear PKM2‐mediated M1 macrophage polarization, while HMW‐HA inhibited the expression of ALPK1 and M1 macrophage polarization.

## AUTHOR CONTRIBUTIONS

**Jie Zhao:** Conceptualization (equal); data curation (equal); formal analysis (equal); methodology (equal); resources (equal); validation (equal); visualization (equal); writing – original draft (equal); writing – review and editing (equal). **Yaping Feng:** Conceptualization (equal); data curation (equal); formal analysis (equal); funding acquisition (equal); supervision (equal); validation (equal); visualization (equal); writing – original draft (equal); writing – review and editing (equal). **Xin Liu:** Data curation (equal); formal analysis (equal); methodology (equal); writing – original draft (equal). **Huimin Li:** Funding acquisition (equal); methodology (equal); writing – review and editing (equal). **Huilin Guo:** Methodology (equal); writing – review and editing (equal). **Jin Ke:** Conceptualization (equal); data curation (equal); methodology (equal); project administration (equal); writing – original draft (equal); writing – review and editing (equal). **Xing Long:** Conceptualization (equal); data curation (equal); funding acquisition (equal); methodology (equal); project administration (equal); supervision (equal); writing – original draft (equal); writing – review and editing (equal).

## CONFLICT OF INTEREST STATEMENT

The authors declared no potential conflicts of interest with respect to the research, authorship and/or publication of this article.

## Supporting information


Figure S1.

## Data Availability

The datasets used and analysed during the current study are available from the corresponding author on reasonable request.
