# Supplementary material for: The relationship of ALPK1, hyaluronic acid and M1 macrophage polarization in the temporomandibular joint synovitis
Source: J Cell Mol Med. 2024 Mar 17;28(7):e18172. doi: 10.1111/jcmm.18172 (PMC10945073; doi:10.1111/jcmm.18172)
Supplement: Supplementary file 1 — Figure S1. [file JCMM-28-e18172-s001.docx]

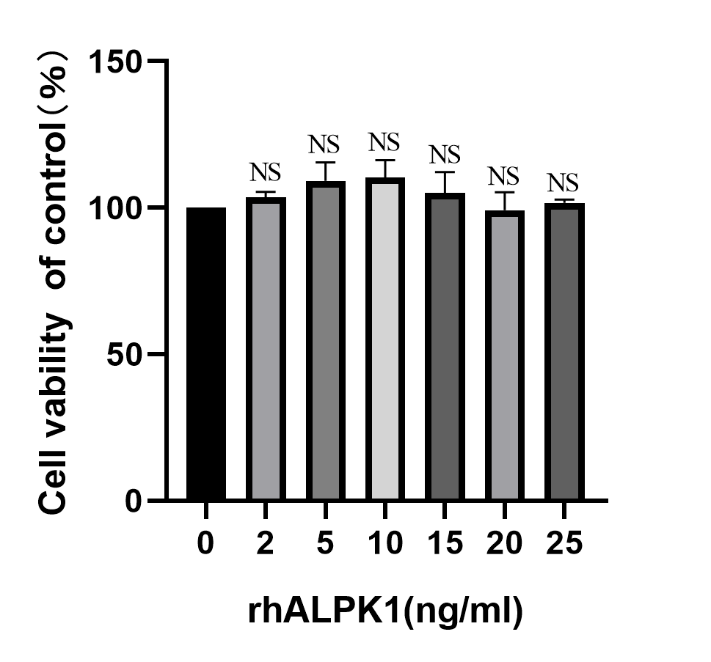


Supplement Figure 1. Effects of rhALPK1 on RAW264.7 cell viability. RAW264.7 cells were treated with rhALPK1 at concentrations of 0, 2, 5, 10, 15, 20 and 25 ng/ml, and cell viability was assessed by CCK-8 assay. One-way analysis of variance, mean ± SEM, n = 4. NS, No Significance compared to control.
